# Practical quantum private query of blocks based on unbalanced-state Bennett-Brassard-1984 quantum-key-distribution protocol

**DOI:** 10.1038/srep07537

**Published:** 2014-12-18

**Authors:** Chun-Yan Wei, Fei Gao, Qiao-Yan Wen, Tian-Yin Wang

**Affiliations:** 1 State Key Laboratory of Networking and Switching Technology, Beijing University of Posts and Telecommunications, Beijing 100876, China; 2 School of Mathematical Science, Luoyang Normal University, Luoyang 471022, China

## Abstract

Until now, the only kind of practical quantum private query (QPQ), quantum-key-distribution (QKD)-based QPQ, focuses on the retrieval of a single bit. In fact, meaningful message is generally composed of multiple adjacent bits (i.e., a multi-bit block). To obtain a message 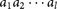 from database, the user Alice has to query *l* times to get each *a_i_*. In this condition, the server Bob could gain Alice's privacy once he obtains the address she queried in any of the *l* queries, since each *a_i_* contributes to the message Alice retrieves. Apparently, the longer the retrieved message is, the worse the user privacy becomes. To solve this problem, via an unbalanced-state technique and based on a variant of multi-level BB84 protocol, we present a protocol for QPQ of blocks, which allows the user to retrieve a multi-bit block from database in one query. Our protocol is somewhat like the high-dimension version of the first QKD-based QPQ protocol proposed by Jacobi *et al.*, but some nontrivial modifications are necessary.

Private information retrieval (PIR), introduced by Chor *et al.*^1^, allows a user (say Alice) to retrieve a bit *x_i_* from a database 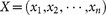 held by a server (say Bob), without revealing the retrieval address *i* (user privacy). A symmetrically private information retrieval (SPIR)^2^ scheme is a PIR scheme satisfying an additional requirement named “database security”, that is, Alice should not get more information than *x_i_* from database. In recent years, many SPIR protocols have been proposed in classical cryptography. However, the security of most classical cryptosystems is based on the assumptions of computational complexity which might be vulnerable to quantum computation^3^,^4^. One may want to know whether this drawback can be overcome by quantum protocols as that in quantum key distribution (QKD)^5^.

In fact, since user privacy and database security appear to be conflicting, the task of SPIR cannot be realized ideally even in quantum cryptography^6^. More practically, the quantum scheme for SPIR problem, called quantum private query (QPQ)^7^, loosens the security into the following. (1) (Database security) Alice can elicit a few more bits than the ideal requirement (i.e., just 1 bit) from database, and (2) (user privacy) user privacy is guarded in the sense of cheat-sensitivity (that is, Bob's attack will be discovered by Alice with a nonzero probability if he tries to obtain Alice's retrieval address), and it would be better if the probability for Bob to reveal the address Alice queries can be kept small meanwhile.

Most earlier QPQ protocols^7^,^8^,^9^ utilizing unitary operations, show great significance in theory, but are difficult to be implemented when a large database is concerned. In 2011, Jakobi *et al.*^10^ proposed a QPQ protocol (J-protocol) based on SARG04 QKD scheme^11^. As many QKD protocols have been realized experimentally, QKD-based quantum private query is more practical and hence has attracted a great deal of concern. In 2012, Gao *et al.*^12^ presented a flexible generalization of J-protocol. Afterwards, Panduranga Rao *et al.*^13^ also gave two modifications of J-protocol's postprocessing. Recently, Zhang *et al.*^14^ designed a QPQ protocol based on a novel counterfactual QKD protocol.

However, the queried message is generally supposed to be a single bit in the above practical QPQ protocols, which is not the fact in a real implementation. In fact, meaningful message is generally composed of multiple adjacent bits, hence Alice has to query many times from database to obtain the entire message in the bit-by-bit way. Here, we turn to a more realistic model called “QPQ of blocks” (QPQB), which allows the user to retrieve a multi-bit block (i.e., multiple adjacent bits) from database in one query. In our QPQB model, for the sake of simplicity, the database *X* is partitioned into entries (blocks) with the same length *l*. Concretely, 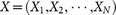, and each entry *X_k_* (1 ≤ *k* ≤ *N*) is an *l*-bit message. Here, *N* is the number of entries in database, and *k* is the address of the entry *X_k_*.

It is worth noting that, the idea of “QPQ of blocks” is natural but nontrivial, since the security of single-bit QPQ cannot be achieved as ideally as that of QKD with the composable security definition^15^,^16^ (e.g., Bob always has a nonzero probability to reveal Alice's retrieval address). Concretely, suppose the database stores blocks of information with the same length of 100 bits and the total number of blocks is 100, then there are 10, 000 bits information in total. If Alice wants the information of the 14th block which contains the bits from 1401st to 1500th, then she has to make 100 queries to obtain these bits in the single-bit QPQ scenario. While as we know, Bob always has a nonzero probability *p* (though it might be very small) to reveal the retrieval address in each bit query. Obviously, once Bob obtains the address of the queried bit in any one of the 100 queries, he can infer which block Alice is retrieving. That is, the probability with which user privacy keeps secret is only (1 − *p*)^100^ in this condition. Apparently, the security degrades very fast with the size of blocks, which is a significant problem for QPQ in real-world applications. Luckily in QPQ of blocks, Alice can obtain the entire block in one query, and as to user privacy, it only needs to hide the address of the block instead of the addresses of its bits. Hence, similar to that pointed out by Chor *et al.*^17^, remarkable saving is possible by utilizing the block structure, and the research on QPQB may be an interesting and worthwhile work.

To fulfill the task of QPQB, we first review the idea for realizing QKD-based QPQ of single bit. As we know, distributing an **oblivious** key is of vital importance to achieve it^10^. That is, Alice and Bob should share a raw key *K^r^* in the way that (1) Bob knows *K^r^* entirely, (2) Alice knows only part of its bits, and (3) Bob does not know which bits are known to Alice. After some classical postprocessing on the raw key, Alice only knows roughly one bit in the final key *K^f^* and Bob still does not know which bit is known to Alice. Then, the final key is used to encrypt database so that (1) Alice can subsequently recover the bit she queries from the encrypted database with her known bit in *K^f^*, and (2) both user privacy and database security are well protected.

Following the above idea, each *l*-bit entry in QPQB needs to be encrypted by an *l*-bit string (i.e., *l* adjacent bits) which should be (1) completely known or unknown to Alice, and (2) completely known to Bob while he does not know whether it is known to Alice. Intuitively, we need to design a *d*( = 2*^l^*)-level oblivious QKD protocol in which transmitting one qudit can provide *l* adjacent bits satisfying the above two requirements. Naturally, we expect that it can be achieved by generalizing the SARG04 protocol^11^ on which J-protocol^10^ is based to its *d*-level version. However, it is scarcely possible. Concretely in the SARG04 protocol, the fact that (1) each key bit is encoded on the basis of the qubit (that is, |0〉 and |1〉 represent bit 0, while |+〉 and |−〉 represent bit 1), and (2) only two complementary bases can be exploited owing to its decipher method, makes it can only generate one bit in the raw key by transmitting one carrier state of any dimension. That is, SARG04 protocol which can be used to generate an oblivious key, cannot be generalized to the high-dimension version. Oppositely, as we know, BB84 protocol^18^ can be generalized to the high-dimension versions^19^,^20^,^21^, but they cannot be used to distribute oblivious key since they are vulnerable to the quantum memory attack^10^. Then, how to overcome this barrier?

In this paper, via an unbalanced-state technique, we design a new QKD scheme which is indeed an intermediate of BB84 and SARG04 protocols. It can not only be used to generate oblivious key, but also be generalized to its high-dimension version (detailed analysis is shown in Methods). On this basis, we propose a quantum protocol for QPQ of blocks, in which the database security is guarded by the impossibility of reliably distinguishing non-orthogonal states, while user privacy is protected by the fact that the states with identical support cannot be unambiguously discriminated. Moreover, our protocol is cheat-sensitive and loss-tolerant.

## Results

Here, we give a quantum protocol for QPQ of blocks, which allows the user to retrieve an *l*-bit entry from database in one query. Our protocol is based on a variant of multi-level BB84 protocol in which the carrier states are transmitted with different probabilities.

### Proposed protocol for QPQ of blocks

**Figure 1:**
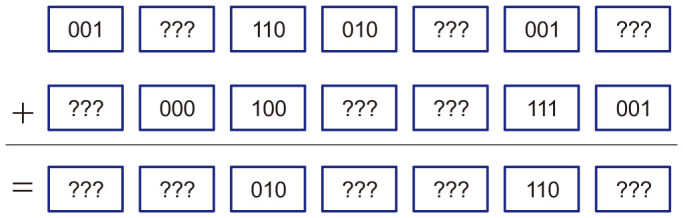
(Gao): Bitwise adding (taking *l* = 3 for example) — an adequate classical postprocessing to reduce Alice's knowledge on the final key. Clearly, Alice's information on the sum string is lower than that on the initial strings. Question marks symbolize Alice's unknown bits.

Let *d* = 2*^l^*, then 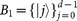 and 
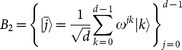
 are two complementary orthogonal bases for *d*-level quantum system, where 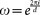. The carrier states adopted in our protocol are chosen from the bases *B*_1_ and *B*_2_, and 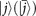 represents an *l*-bit string, i.e., the binary representation of *j*. A detailed description of the protocol is as follows:

(R1) Alice sends Bob a long sequence of qudits which are chosen from basis *B*_1_ or *B*_2_, and among them, each state in 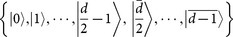 is prepared with probability 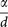, while each in 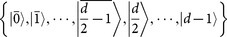 is prepared with probability 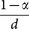. Here, 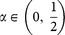.

(R2) Bob measures each received qudit in basis *B*_1_ or *B*_2_ randomly.

(R3) Bob announces in which instances he has successfully detected the qudits; the ones which are not detected are discarded.

(R4) Bob chooses some positions randomly and requires Alice to announce the states of the transmitted qudits there. Then he discards his outputs which are obtained by measuring qudits in incompatible bases, and compares the remaining ones with Alice's announcement. If the error rate is higher than a certain threshold value, or the proportions of the states 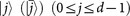 do not coincide with the corresponding probabilities with which they should be prepared in step (R1), the protocol terminates.

(R5) Bob announces all measurement bases he chose in step (R2).

(R6) After dropping the checking qudits, Alice and Bob share an oblivious raw key *K^r^* successfully. Concretely, each element in *K^r^* is corresponding to one measurement result of Bob and hence is an *l*-bit string entirely known to Bob. Apparently, Alice would know half of the elements in *K^r^* by checking the measurement bases announced by Bob. It is worth noting that the raw key is determined by the receiver Bob's measurement outputs rather than Alice's state preparation, which is quite different from previous protocols.

(R7) Enough qudits should be transmitted so that the number of elements in *K^r^* equals to *kN* (*k* is a security parameter, and we will discuss its value later). The raw key is cut into *k* substrings in the way that each substring has *N* elements. These substrings are added bitwise (see Fig. 1) to obtain the final key *K^f^*, and Alice's information on *K^f^* is reduced to roughly one element after that. This process is similar to that in Ref. 10.

(R8) If Alice does not know any element in *K^f^* finally, the protocol fails.

(R9) Suppose that Alice knows the *m*th element 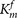 in *K^f^* and wants the *n*th entry *X_n_* in database, she announces the number *s* = *m* − *n*. Then Bob encrypts the database by bitwise adding *K^f^*, shifted by s elements, and sends the encrypted database to Alice. Obviously, *X_n_* is encrypted by 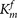 and consequently can be correctly recovered by Alice.

### Features of our protocol

Our protocol is somewhat like the high-dimension version of J-protocol, but some nontrivial modifications are necessary. On one hand, the oblivious raw key in J-protocol is determined by the qudit sender's state preparation, but in our protocol it is determined by the receiver Bob's measurement results (see step (R6)) and hence is entirely known to Bob. On the other hand, the raw key bits are encoded onto the states of the qudits in our protocol while they are encoded onto the bases of the qudits in J-protocol. For these reasons, our protocol can not only resist the quantum memory attack by Bob, but also distribute *l* adjacent bits in *K^r^* by transmitting one qudit, which ensures the realization of “QPQ of blocks”.

Our protocol is loss-tolerant. Note that the qudits in 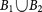 are linearly dependent and cannot be unambiguously discriminated by Bob^22^,^23^. Furthermore, Alice never declares the correct measurement bases in our protocol. It means that Bob cannot make sure the state (or basis) of the qudit by any method. Therefore even in the shield of channel loss, the information Bob could obtain is inconclusive and it would be subsequently compressed in the bitwise-adding phase. Hence, Bob cannot cheat by lying in step (R3) (i.e., announcing that a qudit is lost when he gets an unwanted result) to obtain virtual benefit.

Following the protocol, Alice will know on average 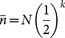 elements in *K^f^* after step (R7). And *P*_0_, the probability that she does not know any element at all and the protocol fails, is 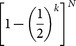. By choosing an appropriate value of *k*, we can ensure both 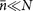 and small *P*_0_ (see Table 1), which implies a successful execution of the protocol. For example, for a database with 10^5^ entries, *k* = 15 is an appropriate choice which provides Alice with 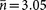 known elements in the final key on average, whereas the probability of failure is only about 4.7%. On the other hand, even if Alice knows 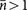 elements in *K^f^*, she can only obtain one chosen entry of the database, because the other 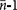 entries known to her will be at random positions in the database.

Now, we study some general attacks and analyze the security of our protocol.

### Database security

To elicit more entries from database, Alice has to know more elements (i.e., Bob's measurement outputs) in the raw key *K^r^*. For this purpose, Alice generally prepares bipartite entangled states |Ψ〉*_AB_*, keeps systems *A* by herself, and sends systems *B* to Bob in step (R1). Then after Bob announces the measurement bases, Alice infers Bob's measurement results by measuring corresponding systems *A*. Without loss of generality, we assume that|Ψ〉*_AB_* can be expressed as


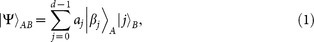



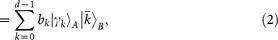


where |*j*〉 ∈ *B*_1_ and 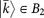.

Let's discuss the conditions for Alice to pass Bob's checking. When being requested to declare the state of one qudit in step (R4), Alice first measure corresponding system *A*, i.e., discriminating 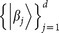 or 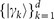 randomly. If the measurement result is |*β_j_*〉 (|*γ_k_*〉), she announces 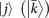 to Bob. To give correct qudit state, system *A* need to be discriminated perfectly no matter which basis Bob chooses, that is, the following conditions must hold.

(i) 〈*β_j_*|*β_k_*〉 = *δ_jk_*, for 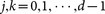.

(ii) 〈*γ_j_*|*γ_k_*〉 = *δ_jk_*, for 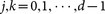.

Meanwhile, to satisfy the required proportions of the qudits, the following conditions must hold.

(iii) 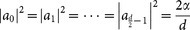, 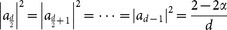.

(iv) 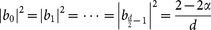, 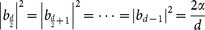.

Since 
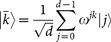
, equation (2) can be written as


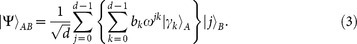


If conditions (ii) and (iv) hold, by comparing equation (1) with equation (3), we have


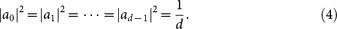


It is clearly contradict with condition (iii). In other words, entangled state which satisfies the above four conditions simultaneously is nonexistent. To avoid being detected, at least two entangled states are needed. One satisfies conditions (i) and (iii) (corresponding to the situation that the carrier states are chosen from *B*_1_), the other satisfies conditions (ii) and (iv).

Therefore, Alice can prepare a long sequence of entangled states which are randomly in state


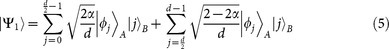


or


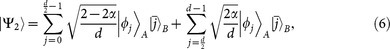


where 〈*ϕ_j_*|*ϕ_k_*〉 = *δ_jk_*, and sends systems *B* to Bob in step (R1) while keeping systems *A* by herself. To announce the state of one qudit correctly in step (R4), she first measures corresponding system *A* in basis 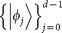. If the measurement result is |*ϕ_j_*〉 and she prepares |Ψ_1_〉 (|Ψ_2_〉) in this position, she announces 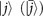 to Bob. Clearly, this kind of attack cannot be detected by Bob.

Now, we discuss the maximal information Alice could gain by this attack. Without loss of generality, we suppose that Alice prepares |Ψ_2_〉 in some position. Then, she can select different strategies to obtain Bob's measurement result after step (R5). If the basis Bob announced in step (R5) is *B*_2_ (which appears with probability 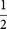), Alice measures system *A* in basis 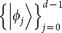 and hence gets Bob's output completely (see equation(6)). If the basis announced by Bob is *B*_1_ (which also appears with probability 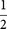), since 
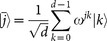
, we have


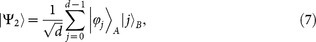


where 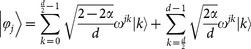. Hence, system *A* would randomly collapse to one of the linearly independent symmetric states 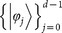 when Bob measures system *B* in basis *B*_1_. Therefore, to infer Bob's measurement result, Alice need to make an unambiguous discrimination on the symmetric states 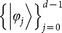
24 with the maximum average success probability being 
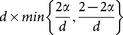
, i.e., 2*α*. Consequently, Alice can obtain at most 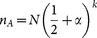 entries from database by this means. When *α* is small, Alice's advantage decreases distinctly. Moreover, with the growth of database size *N*, the ratio 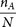 which represents the percentage of the entries Alice would obtain from database, decreases rapidly (see Table 2). Take *α* = 0.1, *N* = 10^5^ for example, dishonest user can get at most 40 entries which occupy only 0.05% of the total entries. It is very little relation to database security for such a complex attack.

**Figure 2:**
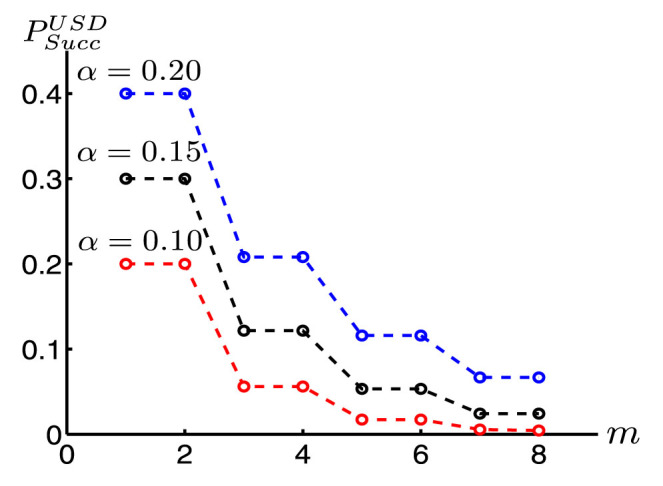
(Gao): For *d* = 2, the maximal success probability 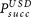 of Alice's joint unambiguous state discrimination (USD) on *m* systems declines rapidly with the increase of *m*.

Now, we consider a more general attack. For those positions where Alice prepares |Ψ_1_〉 (|Ψ_2_〉) while Bob's measurement basis is *B*_2_ (*B*_1_), Alice can postpone the measurement on corresponding systems A held by herself until the very end of the protocol, so that she can know which of them contribute to an element in the final key *K^f^*. Then she can perform a joint measurement on them to guess the final added value in *K^f^* in the way similar to that in Refs. 10, 12. The maximal success probability of Alice's joint unambiguous state discrimination (USD) measurement on *m* systems declines rapidly with the increase of *m* even in the simplest situation when *d* = 2 (see Fig. 2), which means a high security degree for the database security under this kind of attack.

### User privacy

If Bob is dishonest and wants to reveal the address Alice is retrieving, he has to make clear the question whether the measurement basis announced by himself is coincide with the basis of the qudit (i.e., whether the corresponding element in *K^r^* is conclusive in Alice's view) for each received qudit. Therefore, he has to devote himself to judging which basis the qudit is chosen from, i.e., discriminating two equally likely mixed states


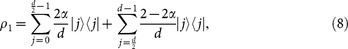


and


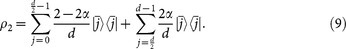


*ρ*_1_ and *ρ*_2_ cannot be unambiguously discriminated because they have the same support^22^,^23^,^25^. However, the protocol is not perfectly concealing because *ρ*_1_ ≠ *ρ*_2_. Bob can make a minimal error discrimination (MED) on them, with the minimal error probability *P_E_*^26^ being


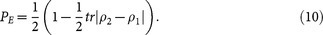


**Figure 3:**
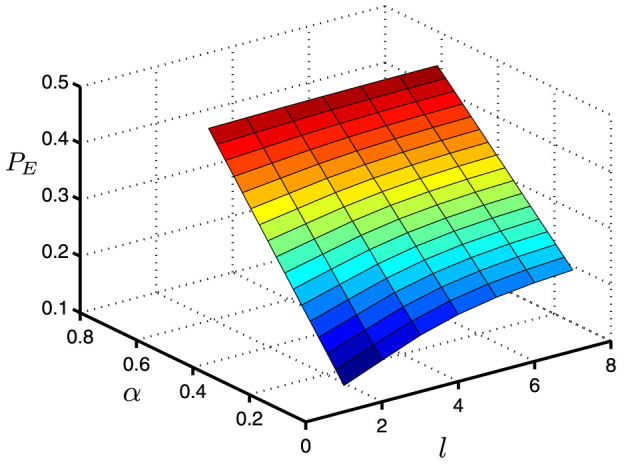
(Gao): The influence of parameter *α* and block length *l* on *P_E_*. Here, *P_E_* is the minimal error probability of Bob's minimal error discrimination on each qudit.

By simple computation, we find that *q_st_*, the element in the *s*th row and *t*th column of matrix *ρ*_2_-*ρ*_1_, satisfies


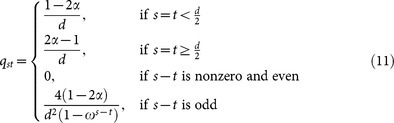


for 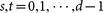. To keep things straightforward, we depict the relationship between *P_E_* and *α*, *l* in Fig. 3. Obviously, the minimal error probability *P_E_* increases with the growth of *l* and *α*. Even in the most favorable situation to Bob where *l* = 1 and *α* is very close to zero, he would make a mistake in the MED measurement on each received qudit with a probability no less than 14.64%. Obviously, it is very difficult for Bob to get Alice's privacy after the bitwise adding phase in step (R7), thus assuring the user privacy in our protocol.

It is worth noting that Bob's attack would be discovered by Alice, because the qudit would be disturbed inevitably in the MED measurement and subsequently Bob could not always output correct value in *K^r^*. Take *d* = 2 for example, the carrier states are chosen from {|0〉, |1〉, |+〉, |−〉}. Here, both |0〉 and |−〉 are prepared with probability 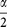, while both |1〉 and |+〉 are prepared with probability 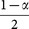. Hence, *ρ*_1_ = *α*|0〉〈0| + (1 − *α*)|1〉〈1|, *ρ*_2_ = (1 − *α*)|+〉〈 + | + *α*|−〉〈−|. The minimal error probability *P_E_* is 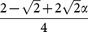, which is larger than 14.64% for all 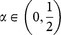, and the MED measurement operators^26^ are Π_1_ = |*ξ*_1_〉〈*ξ*_1_|, Π_2_ = |*ξ*_2_〉〈*ξ*_2_|, where


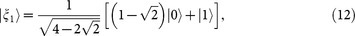



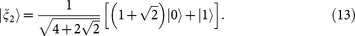


Therefore, the minimal error discrimination of *ρ*_1_ and *ρ*_2_ is equivalent to measuring the received qubit in basis {|*ξ*_1_〉, |*ξ*_2_〉}. Without loss of generality, we suppose that the qubit sent by Alice is |0〉 and corresponding measurement basis announced by Bob is {|0〉, |1〉}, then Bob should output 0 in the generation of raw key to avoid being detected. However, since both |0〉 and |1〉 can collapse to |*ξ*_1_〉 or |*ξ*_2_〉 in the MED measurement (see equations (12,13)), Bob could not output correct result all the time after making the MED measurement on it. His attack would be discovered afterwards when offering false entry to Alice. It indicates that our protocol is also cheat-sensitive.

## Discussion

Compared to QPQ of single bit, QPQ of blocks is not only a more realistic model for application but also a nontrivial idea in security. In this paper, based on a variant of high-dimension BB84 scheme, we propose a protocol to realize QPQ of blocks. Our protocol is cheat-sensitive and loss-tolerant. Besides, the security of our protocol is well protected and the advantages of both sides are strictly limited by *α*. Furthermore, parameter *α* can be changed to balance the advantage between user privacy and database security to satisfy different application requirements. Concretely, in the scenario where the user privacy is emphasized, *α* should be given a larger value; if the database security is more concerned, *α* should be assigned a smaller one. Moreover, in the situation where “fairness to both sides” is pursued, by making a trade-off between user privacy and database security, we can roughly estimate a proper value for *α* (see Supplementary information). From an experimental viewpoint, the *d*-dimension carrier state in our protocol can be prepared with current technology, e.g., a single photon distributed over *d* orthogonal modes as considered in Refs. 27, 28. Recently, some high-dimension BB84-like quantum key distribution protocol has been demonstrated^29^, which also provides fundamental assurance to the application of our protocol.

## Methods

By using a special technique in BB84 protocol, i.e., transmitting the carrier states |0〉, |1〉,|+〉, |−〉 with different probabilities 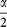, 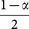, 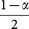, 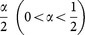 respectively, we obtain an intermediate of BB84 and SARG04 protocols, which can be called unbalanced-state BB84 (US-BB84) protocol (see Table 3). Similar to BB84 protocol, the key bit in US-BB84 protocol is encoded on the state rather than the basis of the qubit. Obviously, the US-BB84 protocol can be generalized to its high-dimension version in the same way as BB84 does^19^,^20^,^21^. Now, we show that it can also be used to distribute an oblivious key as follows:

- (S1) Alice sends Bob a long sequence of qubits, in which *both* |0〉 *and* |−〉 *are prepared with probability* 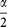
*, while both* |1〉 *and* |+〉 *are prepared with probability* 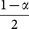. Here the parameter 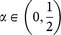. |0〉 and |+〉 represent bit 0, while |1〉 and |−〉 represent 1.
- (S2) Bob measures the received qubits in basis {|0〉, |1〉} or {|+〉, |−〉} randomly.
- (S3) Bob randomly chooses some positions and requires Alice to announce the states of the transmitted qubits there. Then he discards his outputs which are obtained by measuring the qubits in incompatible bases, and compares the remaining ones with Alice's announcement. If the error rate is higher than a certain threshold value, or *the proportions p*(|0〉), *p*(|1〉), *p*(|+〉), *p*(|−〉) *do not coincide with the probabilities* 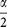, 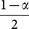, 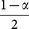, 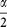, the protocol terminates. Here *p*(|0〉), *p*(|1〉), *p*(|+〉) and *p*(|−〉) represent the proportions of the states |0〉, |1〉, |+〉, |−〉 in Bob's remaining outputs.
- (S4) Bob announces all measurement bases he chose.
- (S5) After dropping the checking qubits, Alice and Bob successfully share an oblivious key *K^r^*, which is composed of Bob's measurement outputs and hence is entirely known to Bob. Obviously, Alice would know half of the bits in *K^r^* by checking the measurement bases announced by Bob.

As shown above, Bob knows the oblivious key entirely, but he cannot reliably infer which bits are known to Alice, because the carrier states are linearly dependent and cannot be unambiguously discriminated. On the other hand, we now show that, owing to the checking of the proportions of carrier states in step (S3), Alice could not obtain the whole key even using entanglement-measurement attack. Generally, Alice can prepare bipartite entangled states in the following forms


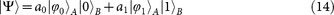



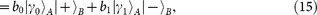


and sends systems *B* to Bob in step (S1). When being requested to declare the state of one qubit in step (S3), Alice first measures system *A*, i.e., discriminating the states 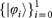 or 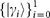 randomly. Then if the measurement result is |*ϕ*_0_〉 (|*ϕ*_1_〉), she announces |0〉 (|1〉) to Bob; if the measurement result is |*γ*_0_〉 (|*γ*_1_〉), she announces |+〉 (|−〉) to Bob. Note that in step (S3), Bob's measurement result would be thrown away if he measures the qubit in basis {|0〉, |1〉} ({|+〉, |−〉}) while Alice announces the state |+〉 or |−〉 (|0〉 or |1〉).

Take *α* = 0.1 for example, to pass Bob's checking in step (S3), Alice has to ensure that (1) she can always declare the states of transmitted qubits correctly, which means that 〈*ϕ*_0_|*ϕ*_1_〉 = 0 and 〈*γ*_0_|*γ*_1_〉 = 0, and (2) after Bob discards the results which are obtained by measuring qubits in incompatible bases, the states |0〉, |1〉, |+〉, |−〉 should occupy 10%, 40%, 40%, 10% of the remaining ones respectively. Therefore, |Ψ〉 must have the following forms


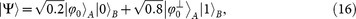



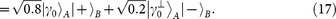


By simple computation, we can find that equations (16) and (17) cannot be satisfied simultaneously. That is, there is no such an entangled state that Alice could pass Bob's checking in step (S3).

However, Alice can prepare a long sequence of entangled states randomly in


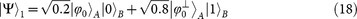


or


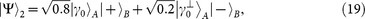


and sends systems *B* to Bob in step (S1). When being asked to announce the state of one qubit in step (S3), if Alice prepared |Ψ〉_1_ there, she measures system *A* in basis {|*ϕ*_0_〉, |*ϕ*_1_〉}, then announces |0〉 (|1〉) to Bob when the measurement output is |*ϕ*_0_〉 (|*ϕ*_1_〉). In this case, Bob would discard his measurement result if he measures this qubit in basis {|+〉, |−〉}. The situation is similar when Alice prepares |Ψ〉_2_. Obviously, Alice can pass Bob's checking in this way.

Then, how many bits can be gained by Alice via this attack? Without loss of generality, we assume that Alice prepares |Ψ〉_1_ and sends system *B* to Bob. If the measurement basis anouced by Bob in step (S4) is {|0〉, |1〉} (which occurs with probability 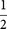), Alice can undoubtedly obtain this key bit by measuring system *A* in basis 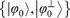 (see equation (18)); if the announced basis is {|+〉, |−〉} (which also occurs with probability 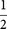), note that |Ψ〉_1_ can also be written as


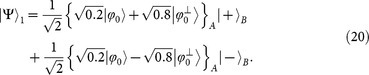


Alice can infer the key bit by unambiguously discriminating the non-orthogonal states 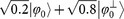 and 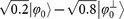 with maximal average success probability being 0.2. That is, Alice can obtain 60% (a little larger than 50% an honest Alice could obtain) of the key bits at most. In fact, Alice could not obtain the whole key as long as 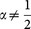.

## Author Contributions

Q.Y.W., F.G. and T.Y.W. analyzed the previous QPQ protocols. All authors designed the new protocol. C.Y.W. and F.G. analyzed its features and security, wrote the main manuscript text and prepared all figures. All authors reviewed the manuscript.

## Supplementary Material

Supplementary Information Supplemental material for “Practical quantum private query of blocks based on unbalanced-state Bennett-Brassard-1984 quantum-key-distribution protocol”

## Figures and Tables

**Table 1:** Possible choices of *k* for different database sizes *N*, as well as the failure probability *P*_0_ and expected number of entries 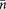 an honest Alice would gain from database

*N*	10^3^	5 × 10^3^	10^4^	5 × 10^4^	10^5^	10^6^	10^8^
*k*	8	11	12	14	15	18	25
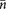	3.91	2.44	2.44	3.05	3.05	3.81	2.98
*P*_0_	0.020	0.087	0.087	0.047	0.047	0.022	0.051

**Table 2:** Alice's advantages for database of different sizes. Here, *α* = 0.1

*N*	10^3^	5 × 10^3^	10^4^	5 × 10^4^	10^5^	10^6^	10^8^
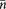	3.91	2.44	2.44	3.05	3.05	3.81	2.98
*n_A_*	16.80	18.14	21.77	39.18	47.02	101.56	284.30
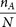	0.0168	0.0036	0.0022	0.0008	0.0005	0.0001	2.8 × 10^−6^

**Table 3:** Comparison of BB84, SARG04 and US-BB84 protocols. The last two columns show that only the high-dimension US-BB84 protocol can be used to realize QPQ of blocks

QKD protocol	state preparation	coding style	generalized to an high-dimension version	used to generate an oblivious key
BB84	randomly chosen (*α* = 0.5)	state	✓	×
SARG04	similar to *α* = 0	basis	×	✓
US-BB84	0 < *α* < 0.5	state	✓	✓
