# Supplementary material for: Practical quantum private query of blocks based on unbalanced-state Bennett-Brassard-1984 quantum-key-distribution protocol
Source: Sci Rep. 2014 Dec 18;4:7537. doi: 10.1038/srep07537 (PMC4269884; doi:10.1038/srep07537)
Supplement: Supplementary Information — Supplemental material for “Practical quantum private query of blocks based on unbalanced-state Bennett-Brassard-1984 quantum-key-distribution protocol” [file srep07537-s1.pdf]

Supplemental material for "Practical quantum private query of blocks based on unbalanced-state Bennett-Brassard-1984 quantum-key-distribution protocol"

Chun-Yan Wei,[1,2] Fei Gao,[1] Qiao-Yan Wen,[1] and Tian-Yin Wang[2]

[1]*State Key Laboratory of Networking and Switching Technology, Beijing University of Posts and Telecommunications, Beijing, 100876, China*

[2]*School of Mathematical Science, Luoyang Normal University, Luoyang, 471022, China*

Parameter and trade-off between two sides

Obviously, parameter α can be changed to balance the advantage between user privacy and database security. Concretely, a smaller α could cut down the number of database entries obtained by a dishonest Alice, while a larger one offers higher security degree of user privacy. Then, to reach fairness for both sides, how to give a proper value of α? Here, we give a rough estimation of this value by making a trade-off between the advantages of the two sides.

We first quantify the advantage of Alice. For a fee-based database with N entries, suppose that the price of each entry is c_1 dollars and the income of Bob is c_2 dollars if he obtains Alice's retrieval address. As showed in section 4.1, Alice can obtain at most $n_A = N(\frac{1}{2}+\alpha)^k$ entries from the database in one query, hence Alice's income function can be defined as

$$Adv_A(\alpha) = c_1[N(\frac{1}{2}+\alpha)^k - 1], \tag{1}$$

which means the maximal extra income the dishonest Alice could gain in one query. Here k is the times of ditwise-adding in step (7).

Now we discuss Bob's income based on information theory. For honest Bob, all of the N addresses are identical, hence the uncertainty (information entropy) of user privacy is $\log(N)$. However, malicious Bob can reduce this uncertainty as follows. After making MED measurement on the received qudits, Bob randomly selects n positions from the N addresses and finds out which qudits contribute to these positions (i.e., which qudits contribute to the dits in these positions in the final key K^f). Then in step (5), he announces the measurement bases of these qudits in a matched way (i.e., if the result of MED measurement is ρ_1 (ρ_2), he announces the measurement basis is B_1 (B_2)). Therefore, for each dit in these n positions in K^f, the probability that it is known to Alice (in Alice's view) is

$$p_1 = (1-P_E)^k. \tag{2}$$

But for those qudits which contribute to the remaining $N-n$ positions, he announces the measurement basis inversely (i.e., if the result of MED measurement is ρ_1 (ρ_2), he announces the measurement bases is B_2 (B_1)). Similarly, for each dit in these positions in K^f, the probability that it is known in Alice's view, can be expressed as

$$p_2 = P_E^k. \tag{3}$$

Therefore, in Alice's view, there would be $n_0 = (N-n)P_E^k + n(1-P_E)^k$ known dits in the final key K^f. Besides, the probability that Alice does not know any dit in K^f and hence the protocol

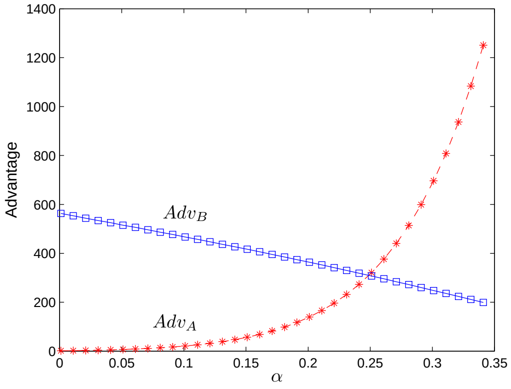

Fig. 1. The advantages of both sides for different α in the situation that $N = 10000, l = 5, \varepsilon = 0.1$, c_1=1, $c_2 = 1000$.

fails, would be $(1-P_E^k)^{N-n}[1-(1-P_E)^k]^n$. Note that P_E, the minimal error probability of Bob's MED measurement on received qudits, is a function of α (see eq(6,7)).

Since $P_E < \frac{1}{2}$, $p_1 \gg p_2$ would usually be satisfied (see eq(11,12)). In other words, the query address is very likely in the n positions Bob selected. Therefore, Bob considers these n positions as the candidates of Alice's retrieval address. Hence, Bob's income will be in two cases: (1) Alice's query address is one of the n positions Bob selected (which appears with probability $p' = n \cdot \frac{(1-P_E)^k}{n_0}$), and the uncertainty of user privacy is successfully reduced to $\log(n)$. Bob's income would be $c_2 \cdot \frac{\log N - \log n}{\log N}$ in this case; (2) Alice's query address is not in the n positions, hence Bob fails and his income is 0. Then Bob's average income is $c_2 \cdot \frac{\log N - \log n}{\log N} \cdot n \cdot \frac{(1-P_E)^k}{n_0}$.

Therefore for a given α, Bob's advantage Adv_B can be defined as Bob's maximum average income under the constraint that, the probability that the protocol fails (i.e., Alice does not know any dit in K^f) is no more than a certain threshold value ε. That is, the maximal average income of Bob $Adv_B(\alpha)$ can be described as

$$Adv_B(\alpha) = \max_{1<n\leq N} \{c_2 \cdot \frac{\log N - \log n}{\log N} \cdot n \cdot \frac{(1-P_E)^k}{n_0}\} \quad (4)$$

$$s.t. \qquad (1-P_E^k)^{N-n} \cdot [1-(1-P_E)^k]^n \leq \varepsilon$$

For any given α, we can obtain $Adv_B(\alpha)$ by going though $n \in [1,N]$ with step size 1 to solve the above problem.

Obviously, the advantages (incomes) of both sides are limited by α (see Fig.4). With the growth of α, Adv_A increases while Adv_B decreases. A proper value of α should realize a trade-off between them, i.e., making Alice's income approximately equal to Bob's. Hence it can be obtained by solving the following problem

$$\min_{0<\alpha<\frac{1}{2}} |Adv_A(\alpha) - Adv_B(\alpha)|. \quad (5)$$

By traveling the parameters $\alpha \in (0, \frac{1}{2})$ with step size 0.001 to solve the above problem, we obtain some proper values of α for different N, c_2 in the situation that l=5, c_1=1 (see Table 3). Obviously, the greater c_2 is, the bigger α becomes (see Fig.5). In other words, if user privacy is valuable, it should be well protected. It is apparently conformity with reality.

Table 1. Proper value of α for different N, c_2 in the situation that $l = 5, c_1 = 1, \varepsilon = 0.1$. Here, l is the length of each entry in database.

	c_2	10	100	1000
$N = 10^3$	α	0.032	0.173	0.330
	Adv_A	5.4164	41.0843	224.2292
	Adv_B	5.4861	40.7933	223.1755
$N = 5 \times 10^3$	α	0.044	0.149	0.271
	Adv_A	5.1739	42.0191	285.1331
	Adv_B	5.2293	41.9104	283.5725
$N = 10^4$	α	0.041	0.137	0.249
	Adv_A	5.2859	43.6348	310.7323
	Adv_B	5.2964	43.4974	312.7566
$N = 5 \times 10^4$	α	0.028	0.110	0.206
	Adv_A	5.5440	48.3842	381.1517
	Adv_B	5.5749	47.8898	379.5053
$N = 10^5$	α	0.026	0.102	0.192
	Adv_A	5.5291	48.4251	398.5687
	Adv_B	5.6136	48.8349	396.0091

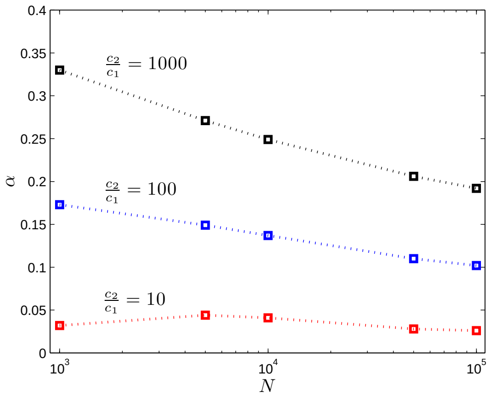

Fig. 2. Proper values of α for different N, c_2/c_1 in the situation that $l = 5, \varepsilon = 0.1$.

Here, we give a rough model to estimate proper value of α from the perspective of fairness. It is undeniable that there are many other methods to estimate this parameter, and some other influences can also be taken into account, e.g., the penalties a dishonest Bob must pay.
